# Parenting profiles: motivation toward health-oriented physical activity and intention to be physically active

**DOI:** 10.1186/s40359-023-01239-7

**Published:** 2023-07-12

**Authors:** Marta Vega-Díaz, Higinio González-García, Carmen De Labra

**Affiliations:** 1 Faculty of Nursing and Podiatry, University of A Coruña (UDC), A Coruña Galicia, Spain; 2 TECNODEF Research Group, Faculty of Education, Universidad Internacional de La Rioja (UNIR), Logroño (La Rioja), Spain; 3 NEUROcom, Centro de Investigaciones Científicas Avanzadas (CICA), Instituto de Investigación Biomédica de A Coruña (INIBIC), Faculty of Nursing and Podiatry, University of A Coruña, A Coruña Galicia, Spain

**Keywords:** Active lifestyle, Adults, Health, Latent profile analysis, Parenting patterns

## Abstract

**Introduction:**

Parents influence their sons’ and daughters’ interest in practicing and maintaining physical activity through parenting patterns.

**Objective:**

To identify perceived parenting style profiles and examine whether the participants differed in their motivation toward health-oriented physical activity and the intention to be physically active.

**Method:**

A sample of 296 participants completed a series of self-report measures and a latent profile analysis (LPA) was performed.

**Results:**

Two profiles emerged as the most suitable: profile (a) with average scores in parenting variables, and profile (b) with high scores in parenting variables. The results revealed significant differences in integrated regulation and in amotivation, reporting higher scores for profile (b) in the parenting variables love/affection, hostility/aggression, and indifference/neglect, and average in undifferentiated/rejection and control.

**Conclusion:**

The combination of perceived parenting style variables in the profiles seems to influence people’s motivation toward health-oriented physical activity. As such, it is crucial to understand parenting from a multivariate approach, mostly in interventions to adjust parenting styles to the most suitable combination.

## Introduction

The American College of Sports Medicine’s stand is that participation in regular physical activity (PA) elicits favorable responses that contribute to healthy aging. PA has demonstrated its beneficial effects in reducing the risk of obesity, diabetes, and cancer [1, 2]. In addition, PA acts positively on mental health [3], cognitive function [4], cardiac and pulmonary function [5], balance [6], gait [7] mobility [8], muscular power, and functional capacity [6]. Based on this evidence, the American College of Sports Medicine recommends that healthy adults perform either aerobic or strength PA [9]. Despite the importance of PA in health, the World Health Organization [10] recognizes that 60% of the population is sedentary. In fact, physical inactivity is one of the biggest public health problems worldwide [11].

Parents are agents that influence their sons’ and daughters’ perceptions of PA to a great extent. Their attitudes toward and respect for sports and PA are essential to children’s behavior [12]. Parenting styles (PS) are a set of educational behaviors that parents communicate to their sons and daughters [13]. PS are especially important in childhood, but their influence continues throughout life [14]. In this study, we use the parental acceptance-rejection theory (PARTheory) [15], a worldwide socialization theory that aims to predict and explain retrospectively the behavior of adults based on the PS perceived during childhood [16]. This may enhance knowledge of parental practices and their influence into adulthood, which may serve to prevent future health problems resulting from parenting. The Spanish version of the PARTheory included five domains: love/affection, hostility/aggression, indifference/neglect, undifferentiated/rejection, and control [17]. Parents can provide love/affection using affectionate gestures and positive comments. Hostility/aggression is present when parents use verbal and physical violence. Indifference/neglect assumes that the parents are not involved in the care of their sons and daughters. Undifferentiated/rejection is manifested by the absence of warm gestures and the deprivation of praise. Finally, control refers to the parents’ degree of supervision over the behavior of their sons and daughters [17]. Other authors have added that control also includes how the mother or father tries to direct the behavior of their sons and daughters with the ultimate goal of them acting in the way that the parent considers desirable [18].

Motivation is a variable that has been examined from multiple perspectives. One is the transcontextual model of autonomous motivation that reflects participation in activities for reasons of choice and volition, and where acting on one’s initiative is considered adaptive behavior [19]. This model maintains that motivation depends on attitudes, subjective norms, and the perceived behavioral control of external agents [20] like parents or teachers. Furthermore, it makes an original contribution to knowledge by illustrating how motivation in one context leads to motivation in another [20]. As far as PA is concerned, if a person performs the activity in physical education classes freely (for the support of parents and teachers), they will probably extrapolate this behavior to their free time. However, this research adopts as a model the self-determination theory (SDT) [21], which also includes contextual agents (parents) but understands motivation as a continuum from intrinsic motivation (the behaviors are carried out autonomously) through extrinsic motivation (external pressures lead to the behaviors being carried out) to amotivation (lack of motivation). Motivation is the internal and external factor that initiates and maintains behaviors [22]. In intrinsic motivation, the behaviors are carried out autonomously; in extrinsic motivation, the behaviors are carried out as a result of external pressures; and in amotivation there a lack of motivation [23].

Previous works have revealed the connection between PS and motivation toward physical activity [21]. In particular, it is known that love/affection creates intrinsic motivation (when people engage in a task for its own pleasure) and extrinsic motivation (when people engage in a task driven by the benefit it will give in the future) [24]. In this case, sons and daughters will participate in PA because they enjoy it, or because they consider that it is optimal for their future. Parental hostility/aggression is externalized by criticism when sons and daughters practice PA. This negative feedback weakens intrinsic motivation [25]. Due to this, sons and daughters will not perceive enjoyment during PA, which may negatively affect their commitment to it [26]. Indifference/neglect is characterized by not meeting the basic needs of sons and daughters, an essential requirement to create motivations with a high degree of integration [27, 28]. For this reason, sons and daughters in this situation do not usually develop motivation for identified and integrated regulation. People who are not motivated by identified regulation do not attribute value to PA, even if it is an activity socially accepted as healthy. People not motivated by integrated regulation do not consider PA part of their values. They do not ascribe value to the PA and do not help others to have an interest in participating in it. Parents who display undifferentiated/rejection do not show affective signals to their sons and daughters; these signals are essential to create intrinsic motivation [24], being the only type of motivation significantly related to PA (at least in adolescent samples) [29]. Because of that, it will be difficult for sons and daughters to perceive enjoyment and commit to PA [26]. Finally, parental control exercised by coercion or punishment generates external regulation or amotivation [30] related to dissatisfaction during PA [31]. This hinders adherence to PA practice, since this happens when perceiving enjoyment [32].

Although the variable intention to be physically active has been previously studied in the field of PA [33], there are only a few studies specifically addressing the topic of PS and the intention to be physically active. Some of the literature postulates that parents who provide their children positive reinforcement (love/affection) help them to strive and engage in the context of physical education [34]. Parents who criticize their sons’ and daughters’ performance during PA favor their perception of stress [35], and stress is a factor linked to the abandonment of physical sports practice [36]. Parents who do not show interest in caring for their sons and daughters do not control or support them (indifference/neglect) [37]; this gives rise to worse levels of moderate to vigorous PA practice (although in a non-statistically significant way) than among parents who provide structure to their sons and daughters [38]. Parental undifferentiated/rejection implies the deprivation of the affectionate signals necessary for sons and daughters to perceive competence in PA [39]. Low perception of competence (efficacy) is a barrier to participation in PA [40]. Finally, high parental control decreases the perception that the actions undertaken emanate from the young people’s own decisions [41], which is usually negatively associated with PA practice [42].

Previous studies examined PS with motivation towards physical activity [24, 28] but did not consider the influence of PS on the intention to be physically active. With our study, we go a step forward, trying to identify the most functional combination of PS variables to promote sons’ and daughters’ motivation toward health-oriented PA and their intention to be physically active. It is relevant to design health intervention programs to include parental education in order to preserve society’s physical wellbeing. Considering that previous works have found that love/affection increases intrinsic motivation [24] but hostility/aggression [25], indifference/neglect [27], undifferentiated/rejection [24], and control [30] weaken motivation with a high degree of integration, and that love/affection increases participation in healthy activities such as sports clubs [43], while hostility/aggression [35], indifference/neglect [38], undifferentiated/rejection [39], and control [42] decrease PA, the starting hypotheses were that profiles that score high in love/affection and low in hostility/aggression, indifference/neglect, undifferentiated/rejection, and control will report high motivations with a high degree of integration and high intention to be physically active, and vice versa. Therefore, the goals of this research were to identify the perceived parenting style profiles and examine whether the participants differed in their motivation toward health-oriented PA and the intention to be physically active.

## Methods

### Participants

The study sample comprised 296 Spanish participants who carry out physical activity (M*age* = 23.26; *SD* = 3.19; 175 men, 121 women). As inclusion criteria, it was selected the Spanish population between 18 and 30 years old (adult population of university age), PA practitioners, who had been raised by both maternal and paternal figures. People from different nationalities instead of the Spanish, underage participants, those over 30 years old, and who adopted sedentary habits were excluded from the study.

### Measures

To evaluate perceived PS it was used the Spanish version [17] of the Parental Acceptance-Rejection Questionnaire (Child PARQ/Control) [44]. The questionnaire consists of 29 items that measure love/affection, hostility/aggression, indifference/neglect, undifferentiated/rejection, and control behavior. The Child PARQ/Control Questions are answered retrospectively by the sons and daughters to know their perception of the maternal and paternal PS. Besides, although the questions related to maternal and paternal figures are identical, the offspring answered them separately to collect information from both the mother and the father. The internal consistency measured by the Cronbach’s alpha is detailed below: love/affection – items 1, 4, 11, 15, 21, 23, 27, 29 - (mother, α = 0.90; father, α = 0.93; eight items, e.g., “My mother/father loves me and needs me”), hostility/aggression – items 5, 8, 12, 17, 22, 24 - (mother, α = 0.88; father, α = 0.91; six items, “My father/mother gets angry and hurts my feelings”), indifference/neglect – items 3, 9, 13, 16, 18, 28 - (mother, α = 0.63; father = 0.68; six items, e.g., “My father/mother ignores me”), undifferentiated/rejection - items 6, 10, 19, 25 - (mother, α = 0.87; father = 0.90; four items, e.g., “My father/mother really does not love me”), and control behavior – items 4, 7, 14, 20, 26 - (mother, α = 0.79; father = 0.80, five items, e.g., “My father/mother wants to control everything I do”). The items are rated on a four-point Likert-type scale, 1 meaning almost never true and 4 meaning almost always true.

Motivation toward health-oriented PA was measured with the Spanish version [45] of Motivation Scale towards Health-oriented Physical Activity (EMAPS) [46] that includes 30 items used to find out the intrinsic motivation - items 1, 8, 16, 24, 27 -, (α = 0.89; five items; e.g., “For the pleasure I feel when I practice PA”), - integrated regulation - items 3, 10, 14, 19, 25 -, (α = 0.85; five items; e.g., “Because PA corresponds to many other aspects of my life”), identified regulation - items 3, 10, 14, 19, 25 -, (α = 0.86; five items, e.g.,“ Because I think PA is good for my personal development”), introjected regulation - items 4, 9, 20, 22, 28 -, (α = 0.80; five items, e.g., “Because I would feel bad if I didn’t make this effort”), external regulation – items 6, 13, 15, 23, 26 -, (α = 0.88; five items, e.g., “Since I don’t have many options, they tell me that I have to do it”), and amotivation – items 2, 7, 11, 18, 30 -, (α = 0.87; 5 items, e.g., “I really do not know; I feel like I’m wasting time when I’m doing PA”) that guide people towards the practice of PA in search of well-being. The responses correspond to a Likert-type scale with a range from 1 (does not correspond at all) to 7 (corresponds very strongly).

To evaluate the intention to be physically active it was used the Spanish version [47] of the Instrument for Measuring the Intention to be Physically Active (MIFA) [48]. The MIFA is composed of 5 items, measuring the intention of being physically active after passing through various educational institutions – items 1, 2, 3, 4, 5-, (α = 0.78; five items, e.g., “After finishing high school, I would like to keep physically active”). The items are rated on a five-point Likert-type scale, 1 meaning totally disagree and to 5 meaning totally agree.

### Procedure

The study was approved by the local Ethics Committee of the International University of La Rioja (UNIR, No. 074/2022) according to the specific national guidelines and conformed to the principles of the Declaration of Helsinki 2013. Before data collection, all of the participants were provided information concerning the study and signed the informed consent. The questionnaire, addressed to the adult offspring, was circulated on Google Online Forms though a link. The questionnaire was structured in four different sections. The first section corresponded to sociodemographic data, the other three to each of the questionnaires used in the study (Child PARQ/Control, EMAPS, and MIFA). The collected data were automatically recorded in Microsoft Excel 365 and later exported to the software package Mplus (v. 7.3) [49] to perform the statistical analysis.

### Data analyses

To perform the latent profile analysis (LPA), Mplus (v. 7.3) software was utilized [49]. An LPA approach was used to identify perceived PS profiles. LPA is a statistical model that stipulates that a variable that cannot be observed (PS) could be inferred through a set of indicators [49]. In the first step, in order to identify the model that best fits the selection of PS profiles, a series of measurement models were carried out until the model that provided the best fit was reached [50]. LPA models are grounded in a series of modeling steps, starting with the specification of a one-class model until there is no further improvement of the model—that is, until the point where adding another class would be meaningless [51]. To ensure that the model follows good fit indices in LPA, there are several statistical indicators. A combination of statistical indicators was used to decide which model fitted the best: log-likelihood value, Akaike information criterion (AIC) [52], Bayesian information criterion (BIC) [53], Adjusted BIC (ABIC) [54], entropy, and the Lo, Mendell, and Rubin likelihood ratio test (LRT) [55]. The model that contains the smallest values on the AIC, BIC, and ABIC and the highest values on the log-likelihood value and the entropy indicates the best fitting model [50]. To examine group differences in PS profiles in motivation toward health-oriented PA and intention to be physically active, we used the Bolck–Croon–Hagenaars method (BCH) [56].

## Results

### Parenting styles profiles analysis

**Fig. 1:**
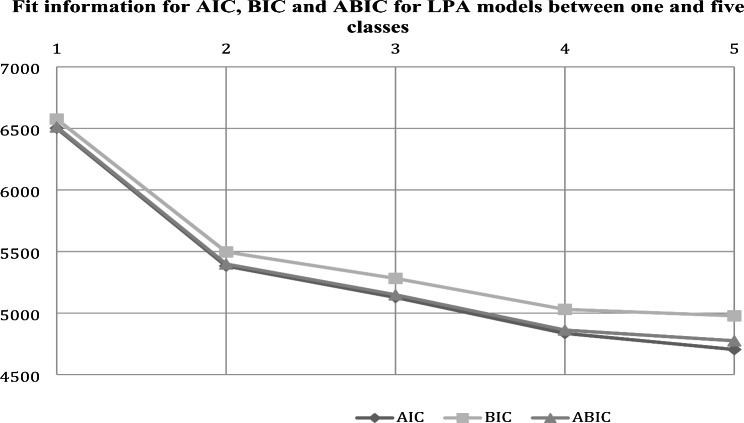
Falls from AIC, BIC and ABIC for LPA models between one and five clases

The LPA models were carried out by initially testing with a one-class model and then exploring models with more than one class up to a total of five classes depending on PS variables (love/affection, hostility/aggression, indifference/neglect, undifferentiated/rejection, and control behavior). For the selection of the best fit profile solution, the values of log-likelihood ratio, AIC, BIC, ABIC, entropy, and LRT were considered. Table 1 includes the fit information (log-likelihood ratio, AIC, BIC, ABIC, entropy, and LRT) for LPA models between the mentioned one to five classes. In this table it can be seen as for AIC, BIC, and ABIC, the biggest drops were recorded between classes one and two (see Fig. 1). The LRTs also found that two classes fit better than one. The highest entropy (0.97) is in profile 2 (together with profiles 4 and 5), however, the most significant p-value continues to be that of profile 2 (*p* = .00). Considering these indices a two-class solution was selected.

**Table 1:** Fit Indices for Latent Profile Analysis Models

No. of classes	1	2	3
No. of free parameters	20	**31**	42
log likelihood	-	**-2,660.40**	-2,521.77
AIC	6,501.63	**5,382.81**	5,127.55
BIC	6,575.10	**5,496.68**	5,281.83
ABIC	6,511.68	**5,398.38**	5,148.64
Entropy	-	**0.97**	0.95
LRT	-	**1,140.82**	277.26
p-value		**0.00** **	0.04*

*Note: AIC = Akaike Information Criterion; BIC = Bayesian Information Criterion; ABIC = Adjusted BIC; LRT = Lo, Mendell, and Rubin Likelihood Ratio Test*

* p < .05; ** p < .001; Bold entries reflect selected model

**Fig. 2:**
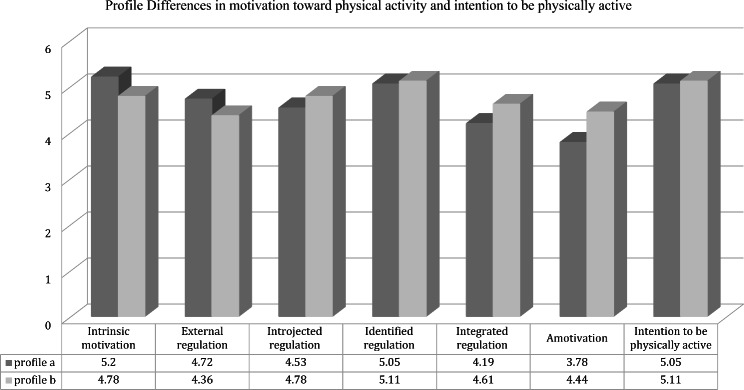
Profile Differences in motivation toward physical activity and intention to be physically active

The Child-PARQ/Control scores were used to differentiate the PS profiles (Table 2). The PS profiles were labeled as: (a) Participants with average scores in love/affection, hostility/aggression, high in indifference/neglect, average in undifferentiated/rejection and control (*n* = 76); (b) Participants with high scores in love/affection, hostility/aggression, indifference/neglect, undifferentiated/rejection, and average in control (*n* = 220) (see Fig. 2).

**Table 2:** Estimates of Latent STAXI-2 Scores and Parenting Styles Profiles for the LPA Model

*Estimates of latent Parenting Styles scores and prevalence of Parenting Styles profiles*	(a) Participants with average scores in maternal/paternal love/affection, hostility/aggression, high in indifference/neglect, average in undifferentiated/rejection and control (*n* = 76)	(b) Participants with high scores in maternal/paternal love/affection, hostility/aggression, indifference/neglect, undifferentiated/rejection, and average in control (*n* = 220)
	*M (SD)*	*M (SD)*
Maternal love/affection	4.29 (0.29)	6.40 (0.44)
Maternal hostility/aggression	4.71 (0.27)	6.22 (0.33)
Maternal indifference/neglect	5.78 (0.23)	5.60 (0.21)
Maternal undifferentiated/rejection	3.91 (0.30)	5.95 (0.47)
Maternal control	2.85 (0.13)	3.06 (0.14)
Paternal love/affection	5.88 (0.44)	10.23 (0.76)
Paternal hostility/aggression	4.39 (0.22)	6.37 (0.29)
Paternal indifference/neglect	6.07 (0.29)	5.38 (0.23)
Paternal undifferentiated/rejection	4.88 (0.38)	8.72 (0.61)
Paternal control	2.98 (0.16)	3.34 (0.19)

### Parenting profiles differences on motivation toward health-oriented PA and intention to be physically active

**Fig. 3:**
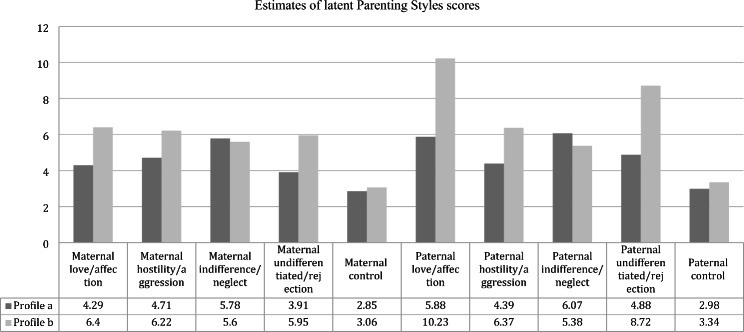
Estimates of latent parenting styles scores

Regarding the parenting profiles differences on motivation toward health-oriented PA, the results of LPA using the BCH method are presented in Table 3, showing significant differences on PS and motivation toward health-oriented PA. In particular, the results showed that profile (b) with high scores in maternal/paternal love/affection, hostility/aggression, indifference/neglect, undifferentiated/rejection, and average in control reported significantly higher scores with respect to the profile (a) with average scores in maternal/paternal love/affection, hostility/aggression, high in indifference/neglect, average in undifferentiated/rejection and control on two out of six dimensions of the EMAPS, being this integrated regulation (*p* = .04) and amotivation (*p* < .00) (see Fig. 3).

**Table 3:** Profile Differences in motivation toward physical activity and intention to be physically active using Bolck Croon, and Hagenaars Method (BCH)

	(a) Participants with average scores in maternal/paternal love/affection, hostility/aggression, high in indifference/neglect, average in undifferentiated/rejection and control (*n* = 76)	(b) Participants with high scores in maternal/paternal love/affection, hostility/aggression, indifference/neglect, undifferentiated/rejection, and average in control (*n* = 220)	
	*M (SD)*	*M (SD)*	*overall test*
Intrinsic motivation	5.20 (0.25)	4.78 (0.14)	0.14
External regulation	4.72 (0.24)	4.36 (0.13)	0.19
Introjected regulation	4.53 (0.18)	4.78 (0.11)	0.24
Identified regulation	5.05 (0.22)	5.11 (0.13)	0.82
Integrated regulation	4.19 (0.17)	4.61 (0.11)	0.04*
Amotivation	3.78 (0.17)	4.44 (0.12)	0.00 **
Intention to be physically active	5.05 (0.22)	5.11 (0.13)	0.83

*Note.*^¥^*p* ≤ .07 * *p* < .05 ** *p* < .01 *** *p* < .001

Regarding the parenting profiles differences on intention to be physically active, the results of LPA using the BCH method are also shown in Table 3; Fig. 3. As it can be seen, there are no statistically significant differences in intention to be physically active between the participants in the two different profiles.

## Discussion

The goal of this research was to identify perceived PS profiles and examine whether the participants differed in their motivation toward health-oriented PA and the intention to be physically active. In this research, it was found that participants differed in motivation toward health-oriented PA (integrated regulation and amotivation), but not in intention to be physically active depending on the combination of perceived love/affection, hostility/aggression, indifference/neglect, undifferentiated/rejection, and control behavior (PS profiles).

In this research, there were two PS profiles: (a) participants with average scores in maternal/paternal love/affection, hostility/aggression, high scores in indifference/neglect, and average scores in undifferentiated/rejection and control; and (b) participants with high scores in maternal/paternal love/affection, hostility/aggression, indifference/neglect, and undifferentiated/rejection, and average scores in control.

Concerning the influence of PS on the motivation toward health-oriented PA, participants from profile (b) with high scores in maternal/paternal love/affection, hostility/aggression, indifference/neglect, and undifferentiated/rejection and average scores in control showed higher integrated regulation and amotivation compared with profile (a) participants with average scores in maternal/paternal love/affection, hostility/aggression, high scores in indifference/neglect, and average scores in undifferentiated/rejection and control.

According to the SDT, in integrated regulation, people perform behaviors of their own free will—that is, the degree of personal self-determination is high [57]. The SDT also postulates that to create motivations with a high degree of integration—that is to say, that the person identifies with a conduct to be carried out and therefore puts it into practice—the primary factor is respect for personal freedom to carry out the behaviors [57]. This aspect is facilitated when parents show love/affection [58, 59]. Profile (b) reports high scores in love/affection. These high scores seem to be responsible for the sons and daughters participating in PA, because it is consistent with their values (integration) [57]. On the other hand, average scores for maternal/paternal control were found in profile (b). The lack of maternal/paternal control may seem like a variable that enhances freedom of action in sons and daughters; however, it has been proven that the existence of a family structure and its boundaries are ideal for guiding the actions of sons and daughters without necessarily inhibiting their freedom [60]. This means that parents of participants from profile (b) will use rules, but they will explain to their sons and daughters the reasons that guide their requests for certain behavior [57]. These parents will not coerce their sons’ and daughters’ freedom of action, and favorable scores in integrated motivation will be maintained [57]. Thanks to this, the integrated regulation will help these participants to consider PA as something inherent to their values. In addition, there are studies that expose that control is associated with low-quality motivation and amotivation, especially if it is exercised coercively, which could explain the motivation that defines profile (b) [30].

In line with that, it should not be ignored that participants from profile (b) reported high scores in maternal/paternal hostility/aggression, indifference/neglect, and undifferentiated/rejection. It was previously reported that parents who gave their sons and daughters negative feedback such as humiliation, insults, and criticism (hostility/aggression) during PA decreased their perception of competence in it [61]. The lack of perceived competence to act makes people not feel motivated [62]. As a result, the sons and daughters will not commit to PA practice [62]. In the same way, parents who express indifference/neglect do not get involved in caring for their sons and daughters, do not control them, do not support them or enhance their self-regulation, and this encourages strong amotivation [63]. Meanwhile, parental undifferentiated/rejection (lack of concern and deprivation of affection toward the sons and daughters) [64] is negatively related to perceptions of efficacy [65]. A low perception of efficacy is related to amotivation [62]. Since the participants of profile (b) perceived greater amotivation than those of the profile (a), they should report a lower commitment to PA [62].

The finding that profile (b) presents high scores in both integrated motivation and amotivation appears contradictory. One possible explanation is that a percentage of the participants from profile (b) are influenced to a greater extent by maternal/paternal love/affection and control despite having maternal/paternal hostility/aggression, indifference/neglect and undifferentiated/rejection, which leads to integrated motivation [57, 58]. On the other hand, perhaps in the other percentage of participants from profile (b) the positive action of maternal/paternal love/affection and control is counteracted by the combination of maternal/paternal hostility/aggression, indifference/neglect, and undifferentiated/rejection, which leads the participants to perceive high amotivation [24, 25, 27, 28].

Regarding the intention to be physically active, it has been possible to verify that there are no significant differences between the participants from profile (a) with average scores in maternal/paternal love/affection, hostility/aggression, high scores in indifference/neglect, and average scores in undifferentiated/rejection and control, and those from profile (b) participants with high scores in maternal/paternal love/affection, hostility/aggression, indifference/neglect, and undifferentiated/rejection, and average scores in control. Despite this, there seems to be a greater intention to be physically active among the participants in profile (b). Profile (b) comprises participants who perceive greater maternal/paternal love/affection than profile (a). Previous research has found that parental love/affection increases participation in healthy activities such as sports clubs [43]. This happens because positive words facilitate the perception of PA competence [66].

Profile (b) also has higher scores than profile (a) in maternal/paternal hostility/aggression, indifference/neglect, and undifferentiated/rejection. PA decreases when parents make use of verbal hostility/aggression. This is because the criticisms that parents make during their sons’ and daughters’ PA performance favor the youngsters’ perceptions of stress and pressure [35]. Parents who use indifference/neglect do not control their sons and daughters and do not support them in their decisions [37], and this seems to relate to worse levels of moderate-vigorous PA practice than among offspring of parents who provide structure [38]. On the other hand, parental undifferentiated/rejection prevents sons and daughters from perceiving efficacy in their actions, because parents deprive them of the signals of affection necessary to perceive competence in PA [39]. Considering oneself competent in PA facilitates the perception of enjoyment during its performance [40], a variable linked to participation [67].

Finally, profile (b) participants have slightly higher scores than profile (a) participants in maternal/paternal control behavior. Parental control decreases the perception that the actions undertaken emanate from the sons’ and daughters’ own decisions [41], which is usually negatively associated with PA practice [42]. Considering the above, profile (b)’s high scores for maternal/paternal hostility/aggression, indifference/neglect, undifferentiated/rejection, and average scores for control, should lead to lower intention to be physically active than in profile (a). Since the results obtained (although not statistically significant) are the reverse, the variable that seems to have the greatest influence on the intention to be physically active among the participants of profile (b) is love/affection. This can be predicted because the love/affection scores are much higher in profile (b) than in profile (a) while the scores are more equal for the other PS variables.

Among the limitations of the research, it is highlighted that the variables examined in this study were evaluated with Spanish adults of university age who were practitioners of PA. The results may not be generalizable to people of other nationalities and different age ranges, and with sedentary behaviors. Moreover, the methodology used is based on data analysis obtained from a self-report questionnaire. Self-report measures may introduce small objectivity biases, such as social desirability or memory biases. Therefore, it would be advisable in future research to include samples of other nationalities, different age groups, and sedentary people to check if the most functional combination of PS dimensions for the future promotion of active lifestyles is the same as that reported in the present investigation. Furthermore, other variables, such as the motivational climate offered by parents, should be examined. Considering the motivational climate that induces concern and the one that leads to sporting success, the motivational climate that induces learning and pleasure during the practice of PA should generate greater future interest in sports practice. Future research could examine how specific behaviors from the SDT perspective apply to intervention programs and seek strategies to increase the intrinsic motivation of sons and daughters. Finally, basic psychological needs could also be examined, since parents influence their sons’ and daughters’ perception of autonomy, competence, and relationships. Besides, this could condition the sons’ and daughters’ participation in PA.

In terms of its practical implications, this work conveys the idea that parents who transmit high love/affection and control at adequate levels favor the motivation of sons and daughters toward health-oriented PA. This research also shows a slight tendency for high love/affection to promote the intention to be physically active. Therefore, parents should offer feedback that shows pride in their sons’ and daughters’ performance in PA, making them feel effective during their practice without ceasing to monitor their participation. As a result, the sons and daughters will integrate the PA as something inherent to their values and participate without being regulated by their parents. This, in turn, will increase their interest in adopting an active lifestyle and preventing active habits from declining over time. Programs that train cognitive skills for positive parenting should consider the results of this research. This would help sons and daughters perceive a combination of appropriate PS variables from their maternal and paternal figures to promote active lifestyles and improve the physical health of adults.

In conclusion, parents influence the motives that lead sons and daughters to initiate new health outreach behaviors and help to maintain them over time. Parents should offer high love/affection and adequate levels of control that facilitate the development of motivations with a high degree of integration toward health-oriented PA in their sons and daughters. In the same way, this love/affection will help to preserve the youngsters’ future interest in the practice of PA. Parents should be considered vital agents in interventions that try to improve the health status of young people through the practice of PA. Identifying the best combination of functional PS variables to facilitate sons’ or daughters’ adoption of an active lifestyle will reduce the high rate of physical inactivity and associated health problems in adults.

## Data Availability

Not applicable.

## List of Abbreviations

PA: Physical activity
PS: Parenting styles
MPA: Motivation towards physical activity
